# Transcriptional Regulation of the Outer Membrane Protein A in *Acinetobacter baumannii*

**DOI:** 10.3390/microorganisms8050706

**Published:** 2020-05-11

**Authors:** Kyu-Wan Oh, Kyeongmin Kim, Md. Maidul Islam, Hye-Won Jung, Daejin Lim, Je Chul Lee, Minsang Shin

**Affiliations:** 1 Department of Microbiology, School of Medicine, Kyungpook National University, 680 Gukchaebosang-ro, Jung-gu, Daegu 41944, Korea; okwan0609@knu.ac.kr (K.-W.O.); horizon112@naver.com (K.K.); maidulbau6923@gmail.com (M.M.I.); jhw921011@naver.com (H.-W.J.); jclee@knu.ac.kr (J.C.L.); 2 Department of Microbiology, School of Medicine, Chonnam National University, Gwangju 61468, Korea; kumdoman7@hanmail.net

**Keywords:** *Acinetobacter baumannii*, *OmpA*, *A1S_0316*, *H-NS*, transcription regulator

## Abstract

*Acinetobacter baumannii* is known for its virulence in severely ill, hospitalized patients and for exhibiting multidrug resistance. *A. baumannii* infection treatment poses a serious problem in clinical environments. The outer membrane protein A (*OmpA*) of the Acinetobacter genus is involved in bacterial virulence. Regulatory factors of *OmpA* in the post-transcriptional stage have been previously identified. However, the regulatory factors that act before the transcriptional stage remain unclear. We investigated the *A1S_0316* gene that encodes a putative transcription factor for *OmpA* expression in *A. baumannii*. A1S_0316 was purified and examined using size-exclusion chromatography, which revealed that it forms an oligomer. The binding affinity of A1S_0316 to the *OmpA* promoter region was also examined. We compared the binding affinity to the *OmpA* promotor region between A1S_0316 and the AbH-NS protein. A1S_0316 showed higher binding affinity to the *OmpA* promotor region than did H-NS. We examined the regulatory effect of these proteins on *OmpA* expression in *A. baumannii* using real-time qPCR and various in vitro tools. Our results indicated that A1S_0316 acts as an anti-repressor on the promotor region of the *OmpA* gene by inhibiting the binding of the AbH-NS protein. This study was the first demonstration of the transcriptional regulation of *OmpA* expression.

## 1. Introduction

Acinetobacter is a genus of strictly aerobic, Gram-negative coccobacillus that is part of the gammaproteobacteria class. The species of the Acinetobacter genus, which are commonly found in soil, are ubiquitous because of their ability to survive even on dry surfaces [1]. *Acinetobacter baumannii* is the major cause of infections among the Acinetobacter species [2]. Because it is an opportunistic pathogen among humans, *A. baumannii* affects individuals who present an immunodeficient state. *A. baumannii* exhibits resistance to many classes of antibiotics, including chloramphenicol, aminoglycosides, and fluoroquinolones [3]. It is one of the bacteria in the ESKAPE group of pathogens (i.e., *Enterococcus faecium*, *Staphylococcus aureus*, *Klebsiella pneumoniae*, *Acinetobacter baumannii*, *Pseudomonas aeruginosa*, and *Enterobacter* species), which are the major cause of nosocomial infections because of their strong antibiotic resistance [4]. The clinical significance of *A. baumannii* stems not only from its ability to survive antibiotic conditions, but also from the various pathogenic factors that it introduces into the host.

The outer membrane protein A (*OmpA*) is one of the porins that are present in the outer membrane of many enterobacteria. With a copy number of ~100,000 per cell, *OmpA* is an abundant membrane protein [5]. The effects of *OmpA* on bacterial virulence have been well studied. Based on the numerous roles it plays as a virulence factor, together with its high copy number, *OmpA* expression is considered to be highly regulated and controlled by several stimuli [6], including the bacterial growth rate and temperature; moreover, the half-life of the *OmpA* mRNA depends on these two parameters [7]. In fact, a previous study has shown that the longevity and stability of the *OmpA* mRNA stems from the highly folded 50-untranslated region (50-UTR) [6]. The two single-stranded regions located within the 50-UTR (i.e., ss1 and ss2) are targets of RNaseE cleavage; however, the cleaving process halts at ss2 when the ribosome complex occupies the ribosome binding site (RBS). In the case of *Escherichia coli*, studies have found that the Hfq protein and a small RNA, MicA, affect *OmpA* expression when bacterial growth reaches the stationary phase [8]. As the bacterial growth rate slows down, Hfq and MicA bind to the RBS, thus inhibiting the attachment of the ribosome complex to the *OmpA* mRNA [9]. This exposes the ss2 region, leading to RNA decay via RNase activity and the downregulation of *OmpA* [6]. Few studies regarding the regulation of *OmpA* expression have focused on the mechanism occurring in the post-transcriptional phase [9,10,11]. In the case of *A. baumannii*, although it is a critical virulence factor, the mechanism and the regulation of *AbOmpA* expression at the pre-transcriptional level have not been studied thoroughly. Here, we examined the possible pre-transcriptional regulators that interact with the promoter region of *AbOmpA*.

## 2. Materials and Methods

### 2.1. Bacterial Strains, Plasmids, and Culture Conditions

The bacterial strains and plasmids that were used in this study are listed in Supplementary Table S1. *A. baumannii* ATCC 17978 and *E. coli* strains were cultured at 37 °C either in Luria–Bertani (LB) broth or on 1.5% (wt/vol) LB agar plates. After transformation with plasmids, the strains were cultured under antibiotic conditions at the following concentrations: kanamycin, 50 μg/mL; chloramphenicol, 20 μg/mL; ampicillin, 100 μg/mL; trimethoprim, 10 μg/mL; and tetracycline, 10 μg/mL

### 2.2. Construction of the ΔA1S_0316 Mutant Strain

The *A1S_0316*-deleted mutant of *A. baumannii* was constructed via the markerless gene-editing method using pDM4:*A1S_0316*. The DNA sequence of the *A1S_0316* open reading frame (ORF) was identified from the information of *A. baumannii* ATCC 17978 in the NCBI database. As the markerless gene-editing method uses the bacterial feature of homologous recombination, the primer sets were designed to result in the overlap of the sequence upstream of *A1S_0316* (1004 bp), the sequence downstream of *A1S_0316* (887 bp), and the *NptI* fragment (KmR, used for antibiotic selection, 1.2 kb). *SpeI* (Enzynomics Inc., Daejeon, Korea) and *ApaI* (TaKaRa Bio. Inc., Otsu, Japan) restriction enzyme sites were added to the 5′ end of the upstream forward primer and the 3′ end of the NptI reverse primer, respectively. The template used for the upstream and downstream fragments was *A. baumannii* ATCC 17978. The template used for *NptI* was pOH4 [12]. The primers used in this experiment are listed in Supplementary Table S2. The three fragments were first obtained separately by PCR, and then connected by crossover PCR in the following order: SpeI-upstream sequence-downstream sequence-NptI-ApaI. The complete insert DNA strand was digested with *SpeI* and *ApaI* and ligated to pDM4. The final cloned plasmid was confirmed through sequencing analysis. The confirmed cloned plasmid was then inserted into the *E. coli* sm10 strain for conjugation. pDM4:*A1S_0316* was delivered from *E. coli* sm10 λ pir cells (donor cells) to *A. baumannii* ATCC 17978 cells (recipient cells) during the overnight incubation of the bacterial mixture on a fresh LB agar plate at 30 °C. The first selection was performed by collecting the bacterial mix, followed by serial dilution by 10-fold, spreading on a Tri/Kan LB agar plate, and incubation at 37 °C, to select the *A. baumannii* that contained the plasmid. The colonies collected during the first selection were then spread onto a 10% (wt/vol) sucrose LB agar plate, to select the *A. baumannii* that had correctly undergone homologous recombination.

### 2.3. Construction of a ΔA1S_0316 Complementary Strain Using pWH1266:A1S_0316

The complementary strain of Δ*A1S_0316 A. baumannii* was constructed by transforming the Δ*A1S_0316* mutant strain with pWH1266:*A1S_0316* including the promoter. To construct pWH1266:*A1S_0316*, an insert DNA was prepared by PCR. The *A1S_0316* obtained (including the promoter) was digested with *PstI* and *EcoRI* and ligated to pWH1266. The clone was confirmed by sequencing analysis. The confirmed plasmid DNA was then inserted into the *A. baumannii* Δ*A1S_0316* strain via electroporation.

### 2.4. Construction of pB4:A1S_0316

The full-length *A1S_0316* gene was amplified by PCR from *A. baumannii* ATCC 17978 using the forward primer 5′–GGGCGGCGGTGGTGGCGGCATGGCAATTTCAAGTTTTGGC–3′ and reverse primer 5′–GTTCTTCTCCTTTGCGCCCTAGATCTCGCCCTTTTCTGAA–3′, which were designed for ligation-independent cloning [13]. The PCR product was treated with T4 DNA polymerase (New England Biolabs) and inserted into the pB4 vector, which is derived from the pET21a plasmid (Novagen) [14]. The construct was confirmed by DNA sequencing analysis.

### 2.5. Construction of pSA508:AbOmpAp for the In Vitro Transcription Assay

To construct a modified pSA508 for the in vitro transcription assay, the DNA sequence of *AbOmpAp* with ranging from the –213 to the +100 position was amplified by PCR using *A. baumannii* 17978 as the template. The PCR product was digested with *PstI* and *EcoRI* and ligated to pSA508 [15].

### 2.6. Primer Extension Assay

The primer extension analysis was performed according to a procedure described previously [16]. *A. baumannii* ATCC 17978 was grown in 50 mL of LB broth to an OD_600_ of 1.0. Total RNA (30 µg) was reverse transcribed in a 50 µL reaction containing AMV reverse transcriptase, dNTP (5 mM), [γ−^32^P] ATP-labeled primer for the *OmpA* gene, and 10 U of RNasin for 50 min at 37 °C. Ethanol precipitation was used to precipitate nucleic acids. Sequencing analysis was performed using an AccuPower DNA sequencing kit (Bioneer, Daejeon, Korea). The mixture was separated by electrophoresis in an 8 M urea/8% (wt/vol) polyacrylamide sequencing gel (40 cm × 0.4 mm). The labeled nucleic acids were detected on an FLA3000 apparatus (Fuji Instrument, Tokyo, Japan).

### 2.7. DNA Affinity Chromatography

The DNA sequence from position −341 to +144 relative to the *OmpA* transcription start site (TSS) was selected as the experimental sample. The biotinylated DNA fragment was obtained by PCR using a 5′-biotinylated forward primer and non-biotinylated reverse primer. Dynabeads™ M-280 Streptavidin (Thermo Fisher Scientific, Waltham, MA, USA) were mixed with the biotinylated DNA fragment in a 1.5 mL Eppendorf tube (E-tube). The beads were collected using a Promega PolyATract 1000 magnetic stand and washed (resuspended by mild vortexing and collected using the magnetic stand) three times with 2× B/W buffer (10 mM Tris-HCl, pH 7.5, 1 mM EDTA, and 2 mM NaCl).

Whole bacterial proteins were extracted when *A. baumannii* ATCC 17978 had been cultured up to an optical density at 600 nm (OD_600_) of 0.5. The harvested bacteria were washed three times with 10 mL of PBS buffer. The bacteria were shortly sonicated and the lysate was separated by centrifuging at 8000 rpm at 4 °C for 20 min. The supernatant was collected for further experimentation.

To identify transcriptional factors that bound to the *OmpA* promoter region, the bacterial lysate was added to a Dynabeads™ M-280 Streptavidin-fused DNA mixture. The combined sample of lysate and probe DNA was incubated by rolling at RT for 20–30 minutes. The protein–DNA probe complex was collected, and the supernatant was removed. The same steps were repeated until each lysate sample was used. After the final reaction, the protein–DNA probe complex was washed with PBS buffer five times. The eluting steps were performed using an increasing series of NaCl solutions (100, 200, 300, 500, 750, and 1000 mM). The eluate samples were analyzed on Mini-PROTEAN® TGX Stain-Free™ Precast Gels (BIO-RAD, Hercules, CA, USA) and stained using a silver-staining kit (Elpis biotech, Daejeon, Korea). The protein bands were identified by liquid chromatography-tandem mass spectrometry (LC-MS/MS) according to their sizes.

### 2.8. Purification of A1S_0316

The purification steps used for A1S_0316 were as described previously [17]. Briefly, pB4:*A1S_0316* was transformed into the *E*. *coli* BL21(DE3) star strain (Novagen). A1S_0316 was purified by sequential chromatographic steps. The purified protein was > 95% pure, as assessed by Coomassie Blue-stained SDS–PAGE.

### 2.9. Electrophoretic Mobility Shift Assay (EMSA)

To perform EMSA, we prepared PCR fragments of *AbOmpAp* with an estimated size of 331 base pairs (positions –231 to +100 relative to the TSS). The primers used for this PCR are listed in Supplementary Table S3. The end of the amplified PCR product was labeled with [γ−^32^P] ATP using T4 polynucleotide kinase (TaKaRa Bio. Inc., Otsu, Japan). The labeled DNA fragment was mixed with A1S_0316 and/or AbH-NS at a specific concentration. The mixtures were incubated for 5 min at 37 °C, loaded onto a 5% native-PAGE gel (acrylamide:bisacrylamide; 40:1), and electrophoresed at 100 V for 1.5 h. The results were imaged on a Fuji Phosphor Imager.

### 2.10. Real-Time qPCR

To perform real-time qPCR, we extracted whole mRNA from the bacteria in the exponential phase, at OD_600_ of 0.5 using a TaKaRa MiniBEST Universal RNA Extraction kit (TaKaRa Bio. Inc., Otsu, Japan). The mRNAs were used as templates to synthesize cDNAs using a TOPscript™ cDNA synthesis kit (Enzynomics Inc., Daejeon, Korea). The quantification of the mRNA transcription was performed using SYBR Green from TOPreal™ qPCR 2× PreMIX (Enzynomics, Korea) and a StepOnePlus Real-Time PCR System (Thermo Fisher Scientific, Waltham, MA, USA). The relative transcription level was calculated by normalizing the CT values to that of the 16s rRNA and was plotted as ΔΔCt. Each experiment was performed in triplicate.

### 2.11. Western Blotting of the Bacterial Membrane Fraction

To compare the translated *AbOmpA* in four bacterial strains, i.e., *A. baumannii* 17978 WT, Δ*OmpA*, Δ*A1S_0316*, and Δ*A1S_0316*/*p0316*, we collected AbOmpA via the membrane fraction because of its high copy number. The bacteria were cultured toOD_600_ of 0.5. Each bacterial strain was harvested and sonicated for 10 min. The supernatants from the centrifuged samples were collected by ultracentrifugation (Type 70 Ti rotor) at 100,000 × *g* for 1 h. The pellets gathered were each resuspended in 2% sodium lauroyl sarcosinate in hydroxyethyl piperazineethanesulfonic acid (HEPES) buffer. The samples were ultracentrifuged at 100,000 × *g* for 1 h. The pellets were resuspended in 10 mM HEPES buffer. The concentration was measured by measuring the absorbance of the sample at 280 nm (A_280_) using a Nanodrop spectrmeter.

The anti-OmpA antibody was prepared in rabbits. The samples from the membrane fraction of each bacterial strain were loaded onto 10% SDS–PAGE, followed by transfer to a nitrocellulose membrane (GE Healthcare, Amersham, UK) and probed with the anti-OmpA antibody. The bound anti-OmpA antibody was detected using a SuperSignal™ West Pico PLUS Chemiluminescent Substrate kit (ThermoFisher Scientific, Waltham, MA, USA) at a ratio of 1:1 and was visualized on a Fusion FX imaging machine (Vilber, Lamirault, France)

### 2.12. In Vitro Transcription Assay

The procedure used for the transcription assay was carried out according to a method described previously [18]. pSA508:*AbOmpAp* (2 nM), 1 mM ATP, 0.1 mM GTP, 0.1 mM CTP, 0.01 mM UTP, and 10–20 μCi of [α−^32^P] UTP, together with a specific amount of A1S_0316 and AbH-NS, were pre-incubated in the following buffer: 20 mM Tris pH 7.8, 10 mM magnesium acetate, 100 mM potassium glutamate, and 1 mM dithiothreitol) at 37 °C for 5 min. The transcription was started by adding RNAP (20 nM) in a total reaction volume of 20 μL. The reaction was incubated at 37 °C for 10 min and was treated with an equal volume of RNA loading buffer (80% (*v*/*v*) deionized formamide, 1× TBE (89 mM Tris, 89 mM boric acid, and 2 mM EDTA), 0.025% bromophenol blue, and 0.025% xylene cyanole), to terminate the reaction. The mixtures were separated in an 8 M urea/7% polyacrylamide gel via electrophoresis. The result was imaged on a Fuji Phosphor Imager.

## 3. Results

### 3.1. Characterization of the OmpA Promoter Region in A. baumannii

**Figure 1:**
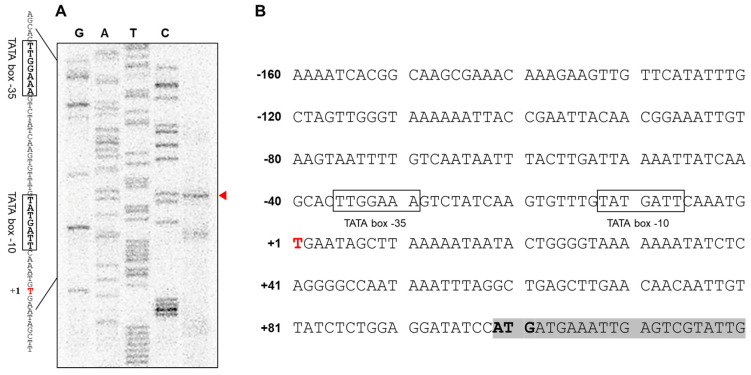
The results of the primer extension assay indicate the location of the transcriptional start site (TSS) of *AbOmpA* (**A**,**B**). To identify the TSS of *Acinetobacter* *baumannii OmpA*, we performed a primer extension assay. The TSS was located at position –89 from the start codon. Putative −35 and −10 region are marked in open box.

To pinpoint the TSS of *AbOmpA*, we performed a primer extension analysis. We found that the TSS was located at the thymine at position –89 from the start codon of *AbOmpA* (Figure 1A). We determined the location of the –35 and –10 TATA boxes according to the location of the TSS (Figure S1). We analyzed the *AbOmpAp* gene sequence by comparing it with the promoter region sequence of *OmpA* from other Gram-negative bacteria using the Multi-Align Program (AMAP). The bacteria chosen for this comparison were *E. coli* MG1655(Ec), *Klebsiella aerogenes* KCTC 2190(Kaer), *K. pneumoniae* HS11286(Kpn), *Shigella flexneri* str. 301(Sfl), and *Salmonella typhimurium* str. 798(Styph) (Figure S1). The location of the TATA boxes and the TSS seemed to be conserved among the six bacterial strains. However, the specific DNA nucleotides of *AbOmpAp*, including its TATA box and TSS, showed an atypical sequence, while the promoter regions of the remaining five bacterial strains showed a strong similarity.

### 3.2. Identification of Proteins that Bind to the OmpA Promoter

**Figure 2:**
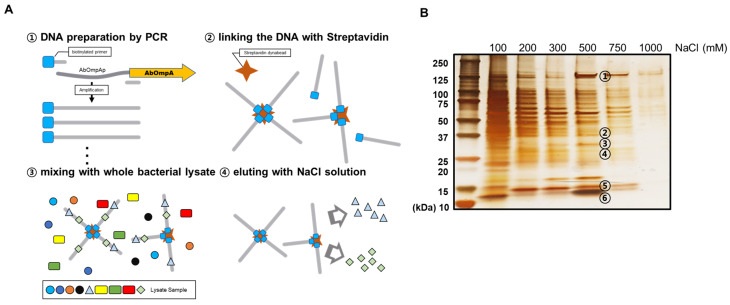
Identification of proteins that bind to the *AbOmpA* promoter. The diagram depicts the overall scheme of the DNA affinity chromatography experiment (**A**). The proteins eluted from the DNA affinity chromatography were loaded onto 4–15% gradient SDS–PAGE and developed via silver staining (**B**). The bands with noticeable thickness in the 500 mM NaCl lane were chosen for identification via LC-MS/MS. The specific information of the identified proteins is listed in Supplementary Table S2.

To identify putative transcriptional regulators of the promoter region of *AbOmpA*, we performed a DNA affinity chromatography analysis. We collected proteins that bound to the *AbOmpA* promoter using the following salt concentrations (100, 200, 300, 500, 750, and 1000 mM NaCl) (Figure 2A). Numerous protein samples were eluted from the chromatography column, which is shown in the silver-stained SDS–PAGE (Figure 2B). Among the eluates, each band from 500 mM NaCl was identified by LC-MS/MS analysis. The RNA polymerase subunits (α-subunit, 34.6 kDa; β-subunit, 150 kDa) were identified and confirmed, indicating that the lysate was properly prepared. We identified a protein (29.3 kDa) expressed by the gene labeled *A1S_0316*, which was classified as a “Putative Transcription Factor” in the NCBI database. We also found that *H-NS* (*AbH-NS*), which is a DNA-binding protein that is well known as a global repressor, was present in the same eluate. Further information about the proteins identified here is provided in Supplementary Table S2.

### 3.3. Molecular Features of A1S_0316

**Figure 3:**
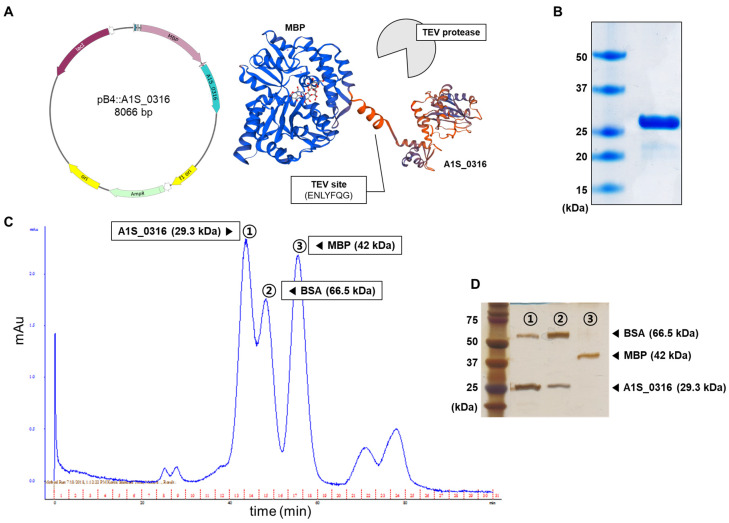
Characterization of the A1S_0316 protein. Overall scheme used for the purification of A1S_0316 (**A**). The purified A1S_0316 was loaded onto 15% SDS–PAGE (**B**). A1S_0316 (29.3 kDa) was compared by size-exclusion chromatography with proteins of different sizes, such as maltose-binding protein (MBP) (42 kDa) and bovine serum albumin (BSA, 66.5 kDa) (**C**). The eluents from the three kinds of high peaks were separated through 15% SDS–PAGE (**D**).

To characterize A1S_0316 as a putative transcription regulator, we purified A1S_0316 by Ni-NTA affinity column. We used the pB4 vector and expressed it as a tag-fused maltose-binding protein (MBP), including the TEV protease site (Figure 3A). The purified A1S_0316 was confirmed by the presence of a 29.3 kDa band on SDS–PAGE (Figure 3B). Moreover, we compared the size of purified A1S_0316 with that of albumin (66.5 kDa) and MBP (48 kDa) via size-exclusion chromatography, in which the larger molecules are eluted faster. We observed three peaks in absorbance, which corresponded to the three samples input by us (Figure 3C). We confirmed the molecules from the three peaks using SDS–PAGE (Figure 3D). The first and second eluents seemed to contain the same types of proteins (albumin and A1S_0316), albeit at different concentrations. The results of a gel filtration analysis led us to conclude that A1S_0316 formed an oligomer.

**Figure 4:**
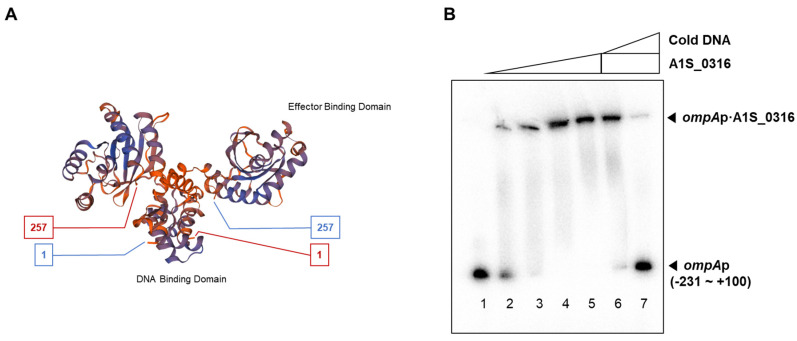
Prediction of the structure of A1S_0316 and specific binding of A1S_0316 to the *AbOmpA* promoter. The structure prediction model of A1S_0316 was obtained by submitting the amino acid sequence of the protein to the SWISS-MODEL program (**A**). The interaction between the purified A1S_0316 and the *AbOmpA promoter* (–231 to +100) was shown by gel shift assay (**B**). In lanes 6–7, “cold DNA” specifies the unlabeled competitor *AbOmpA* promoter DNA fragments used at 10-fold excess relative to the labeled probe DNA.

We also attempted to predict the structure of A1S_0316 using the SWISS-MODEL program and the amino acid sequence from the NCBI database. It is presumed to have an IclR-type helix-turn-helix domain and an IclR effector binding domain (Figure 4A,B). To assess the binding ability of A1S_0316 to the promoter region of *AbOmpA*, we performed a gel shift analysis with purified proteins and the isotope-labeled promoter region. The size of the isotope-labeled *AbOmpA* promoter DNA strand with a certain concentration of A1S_0316 added is examined inside the native-PAGE (Figure 4B). To confirm the specific binding complex of the *AbOmpA* promoter and A1S_0316, we added a non-labeled *AbOmpA* promoter DNA as a competitor. The shifted band from the DNA–protein complex disappeared with an increasing amount of cold-DNA probe (Figure 4B). We concluded that A1S_0316 binds to the promoter region of *AbOmpA* specifically.

### 3.4. Effect of A1S_0316 and AbH-NS on OmpA Expression In Vivo

**Figure 5:**
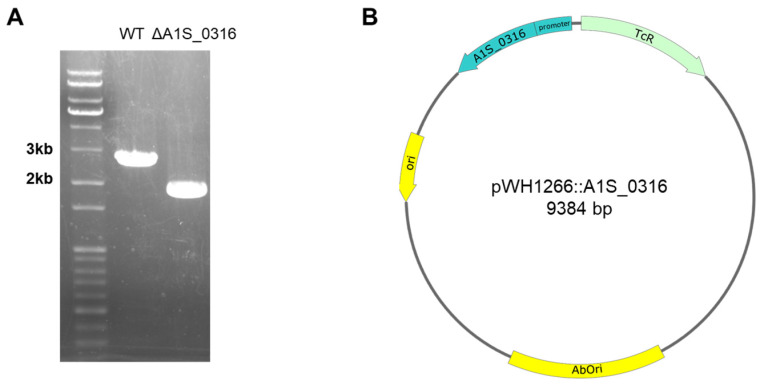
The markerless gene-editing method used for constructing the *A. baumannii* Δ*A1S_0316* mutant (**A**). The DNA-stained 1% agarose gel shows that the length of *A1S_0316* nearby DNA sequence was shorter than that of the wild-type strain (**A**). To complement the mutant strain, pWH1266:*A1S_0316* including its promoter was constructed (**B**).

**Figure 6:**
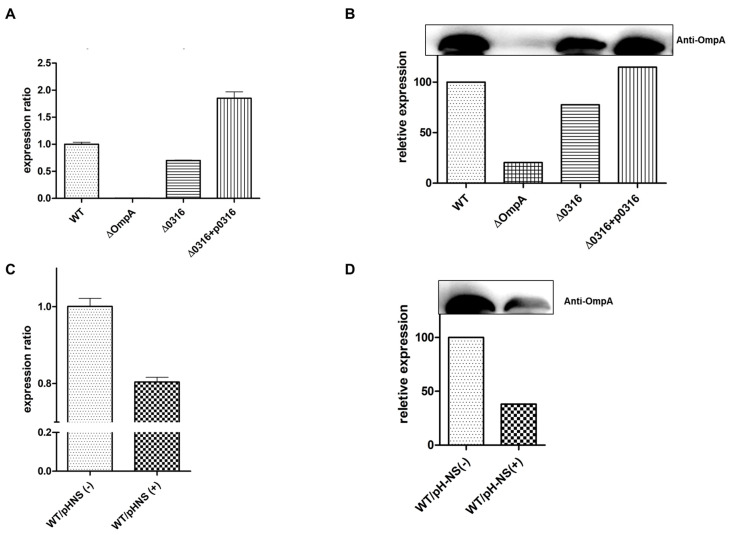
Effect of A1S_0316 and AbH-NS on *AbOmpA* expression in vivo. The differences in the expression of *AbOmpA* at the transcriptional and translational stage are shown according to each condition. The RNA level of *AbOmpA* was determined by real-time qPCR (**A**,**C**). The gels shown in (**B**,**D**) were transferred to nitrocellulose membranes and probed with an anti-AbOmpA antibody. Bound antibodies were detected by enhanced chemiluminescence (ECL). WT/p*H-NS*(+) indicates the addition of 10 mM l-arabinose, for *AbH-NS* induction.

Based on the finding that A1S_0316 bound to the *AbOmpA* promoter, we hypothesized that A1S_0316 is a transcriptional regulator of *AbOmpA* expression. To understand the effect of A1S0316 on *AbOmpA* gene expression, we constructed the *A1S_0316* deletion mutant (Δ*A1S_0316*) in the *A. baumannii* 17978 strain using the markerless gene-editing method (Supplementary Figure S2). We confirmed the *A1S_0316* deletion mutant by PCR (Figure 5A). We also constructed the complementary strain by inserting the ORF of *A1S_0316*, including its promoter region, into the pWH1266 plasmid, which was replicated in the *A. baumannii* strain (Figure 5B). We examined the effect of A1S_0316 on *AbOmpA* expression level by comparing it in the wild-type, Δ*OmpA*, Δ*A1S_0316*, and Δ*A1S_0316*/pWH1266:*A1S_0316* strains by real-time qPCR (Figure 6a). In Δ*A1S_0316*, *AbOmpA* gene expression was decreased 2-fold compared with the wild-type strain. We also examined the levels of the *AbOmpA* protein in the presence or absence of A1S_0316 via Western blotting using an anti-OmpA antibody (Figure 6B). The *AbOmpA* protein was downregulated in Δ*A1S_0316* compared with the wild-type strain. These data led us to conclude that A1S_0316 regulates *AbOmpA* expression as an activator.

To examine the effect of AbH-NS on *AbOmpA* expression, we constructed a plasmid containing the *araBAD* promoter-fused *AbH-NS* ORF, which overexpresses *AbH-NS* using l-arabinose. We transformed pBAD:*AbH-NS* with *A. baumannii* 17978. The expression level of *AbOmpA* was decreased in cells overexpressing *AbH-NS*, as assessed by real-time qPCR and Western blot analysis (Figure 6C,D). These experiments led us to conclude that A1S_0316 affects *AbOmpA* expression as an “activator-like” regulator, while AbH-NS acts as a repressor, which is in accordance with the results of past studies showing that H-NS is a global repressor in Gram-negative bacteria [19].

### 3.5. Effect of A1S_0316 and AbH-NS on OmpA Expression In Vitro

**Figure 7:**
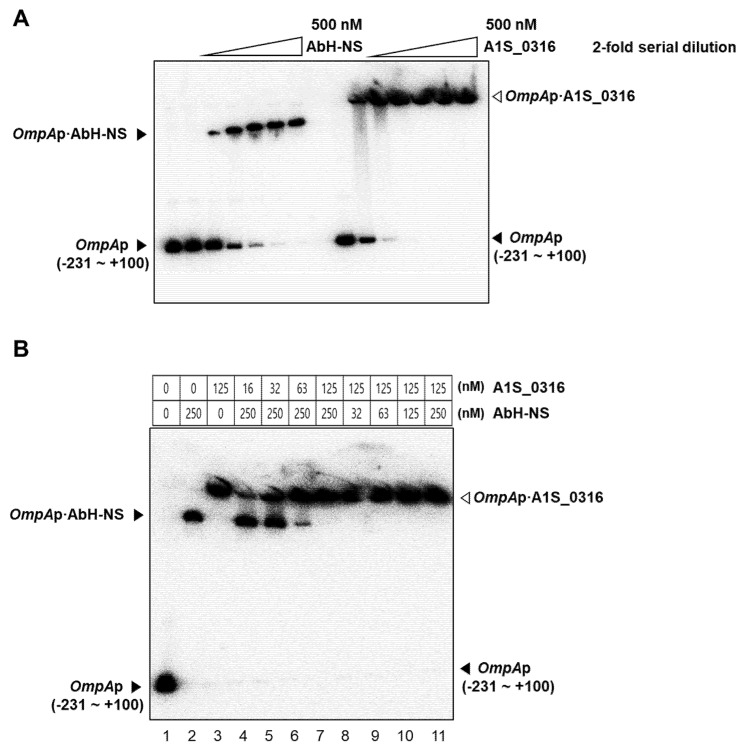
Binding of A1S_0316 and AbH-NS to the *AbOmpA* promoter region. The DNA difference in the affinity to the *AbOmpA* promoter between A1S_0316 and AbH-NS was confirmed by EMSA (**A**). Each protein sample was added in an increasing amount, with the last lane representing 500 nM of each protein (**A**). DNA–protein complexes were detected by gel shifts on 5% native polyacrylamide gels. Lanes 4–7: the *AbOmpAp* DNA was first incubated with *AbH-NS* (250 nM) for 2 min, followed by increasing concentrations of A1S_0316 (16, 32, 63, and 125 nM) for 2 min (**B**). Lanes 8–11: the *AbOmpAp* DNA was first incubated with A1S_0316 (125 nM) for 2 min, followed by increasing concentrations of AbH-NS (32, 63, 125, and 250 nM) for 2 min (**B**). The open arrowhead indicates the *AbOmpAp*–A1S_0316 complex, and the closed arrowhead indicates the *AbOmpA*–AbH-NS complex.

To confirm the effects of A1S_0316 and AbH-NS on *AbOmpA* expression observed in vivo, we performed gel shift and in vitro transcription assays. We tested the binding affinity of both A1S_0316 and AbH-NS to the *AbOmpA* promoter using a gel shift assay. First, we introduced the isotope-labeled *AbOmpAp* with the same molar concentration of AbH-NS and A1S_0316, respectively (Figure 7A). Interestingly, when the two proteins were gradually applied in equal amounts to the labeled *AbOmpA* promoter, A1S_0316 caused the bands to “shift” at a lower concentration than that of AbH-NS. Second, to identify the relationship between the two proteins, we conducted another gel shift assay in which we applied both A1S_0316 and AbH-NS to the *AbOmpA* promoter (Figure 7B). The gradual application of A1S_0316 to the *AbOmpA* promoter at the saturating amount of AbH-NS led to a decrease in the AbH-NS–*AbOmpAp* complex, whereas the A1S_0316–*AbOmpAp* complex remained present. However, the AbH-NS–*AbOmpAp* complex disappeared at the saturating amount of A1S_0316, while the bands presumed to correspond to the A1S_0316–*AbOmpAp* complex remained unchanged (Figure 7B). We concluded that the A1S_0316 binding affinity to the *AbOmpA* promoter region is higher than that of AbH-NS.

**Figure 8:**
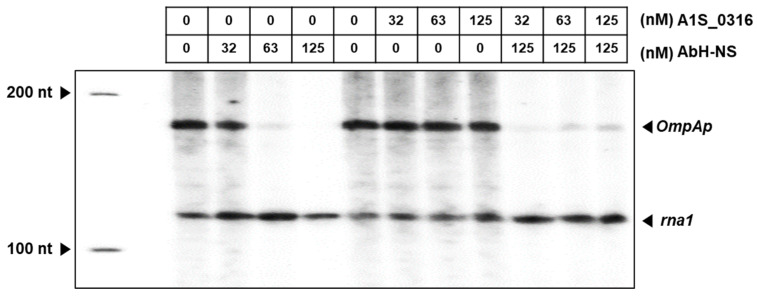
Regulatory effect of A1S_0316 and AbH-NS on *AbOmpAp*. The role of A1S_0316 and AbH-NS in the regulation of the *AbOmpA* promoter was examined in the transcription phase in vitro. This assay was validated by the expression of the rna1 RNA, which was the control derived from the plasmid pSA508, carrying *AbOmpAp* (–231 to +100). The lane on the left corresponds to the 100-nucleotide ladder. The rna1 RNA is 106 nucleotides and *AbOmpA* is 149 nucleotides. The radioactive *AbOmpA* transcripts were analyzed on an 8% sequencing gel.

To assess the relationship between A1S_0316 and AbH-NS at the level of the transcription of *AbOmpA*, we then performed an in vitro transcription assay (Figure 8). The pSA508 plasmid carrying the *AbOmpAp* DNA sequence (positions −213 to +100), followed by the strong rpoC terminator, was used as the template. The results revealed a gel pattern of a multi-round transcription assay. There are two transcripts with distinct lengths detected by *AbOmpAp* RNA and rna1 transcript from the origin of replication as a control. The band corresponding to the *AbOmpA* transcript was decreased when the amount of H-NS was increased. However, in the presence of increasing amounts of A1S_0316, the *AbOmpAp* RNA was not changed, as assessed based on band intensity. Interestingly, the addition of A1S_0316 restored the *AbOmpAp* RNA, which had been repressed at high concentrations of H-NS. These results suggest that A1S_0316 not only binds to the *AbOmpA* promoter but also does so with stronger affinity than does *AbH-NS*, thus blocking the repressing effect of the latter on *AbOmpA* expression.

## 4. Discussion

In this study, we discovered a potential transcription factor for *AbOmpA* termed A1S_0316 and determined the role of this protein among the regulatory mechanisms of this molecule. The scarcity of studies of *AbOmpA* regulation caught our attention because this protein is one of the virulence factors of *A. baumannii*. First, we analyzed the DNA sequence of the *AbOmpA* promoter, to identify the TSS and the TATA boxes used for *AbOmpA* expression (Figure 1A,B). The c*OmpA*rison of the *AbOmpA* promoter based on the analysis of the *OmpA* promoter sequences of other Gram-negative bacteria showed that the sequence located in the upstream region of the *AbOmpA* promoter is unique (Supplementary Figure S1). To identify putative transcriptional regulators on the promoter region of *AbOmpA*, we performed a DNA affinity chromatography analysis. We identified proteins that bound to the *AbOmpA* promoter by LC-MS/MS analysis (Figure 2). Among them, we focused on A1S_0316, which is a putative transcriptional regulator that contains a helix-turn-helix motif at the N terminal and is a member of the iclR family [20]. We assumed that A1S_0316 forms homo-oligomers in its natural state. We then submitted A1S_0316 to size-exclusion chromatography with albumin and MBP to predict the natural form of A1S_0316. The elution speed of purified A1S_0316 was the fastest among the three proteins, indicating that it had the largest size (Figure 3C). We then confirmed the direct binding interaction between the *AbOmpA* promoter and A1S_0316 through a gel shift analysis. A1S_0316 specifically bound to the *AbOmpA* promoter (Figure 4). IclR is involved in intracellular growth, toxicity, and gene regulation of the human pathogenic *F. tularensis* subspecies [21]. We tested growth and biofilm formation using the A1S_0316 deletion strain. No significant changes were observed compared with the wild-type strain (Supplementary Figure S3). To identify the effect of A1S_0316 on *AbOmpA* expression in vivo, we constructed Δ*A1S_0316* via the markerless gene-editing method and transformed this mutant with the pWH1266: *A1S_0316*, to complement the deficient feature (Figure 5). The *A1S_0316*-deficient strain exhibited decreased levels of both transcription and translation, as assessed using Western blotting and q-PCR analysis (Figure 6A,B), which indicates that A1S_0316 plays an activator-like role in the *AbOmpA* expression mechanism. Interestingly, the expression of *AbOmpA* was repressed by AbH-NS in vivo (Figure 6C,D). We then examined the relationship between A1S_0316 and AbH-NS in the mechanism of *AbOmpA* expression. To this end, we compared the binding affinity of each protein to the *AbOmpA* promoter using a gel shift analysis. The results of these EMSAs showed that, in the presence of the same amounts of these proteins, A1S_0316 bound to the DNA more effectively than did AbH-NS, which implies the higher binding affinity of A1S_0316 (Figure 7). Subsequently, we examined the effect of A1S_0316 and AbH-NS on *AbOmpA* expression in vitro, individually and together. The addition of A1S_0316 restored the *AbOmpAp* transcript, which had been repressed in the presence of a high concentration of AbH-NS (Figure 8). In Gram-negative bacteria, H-NS participates in a variety of anti-silencing mechanisms that involve DNA-binding proteins such as Ler, LeuO, RovA, SlyA, and VirB [18,22,23]. We concluded that A1S_0316 acts as an anti-repressor of AbH-NS in the mechanism of *AbOmpA* expression.

The *OmpA* in *A. baumannii* is a very important virulence factor which mediates pathogenesis, biofilm formation and antibiotic resistance [24,25]. In recent years, researchers emphasized the use of *OmpA* as a potential therapeutic target against *A. baumannii* [26]. In this study, we showed for the first time that A1S_0316 could be transcriptional regulator of *AbOmpA* expression, as an anti-repressor. Here, we investigated how *A1S_0316* affects the *AbOmpA* expression mechanism along with other unknown factors. Further study of other factors that cooperate with A1S_0316 is needed to understand the mechanism of *AbOmpA* expression.

## Supplementary Materials

The following are available online at https://www.mdpi.com/2076-2607/8/5/706/s1.
