# Supplementary material for: Transcriptional Regulation of the Outer Membrane Protein A in Acinetobacter baumannii
Source: Microorganisms. 2020 May 11;8(5):706. doi: 10.3390/microorganisms8050706 (PMC7284552; doi:10.3390/microorganisms8050706)
Supplement: Supplementary file 1 [file microorganisms-08-00706-s001.pdf]

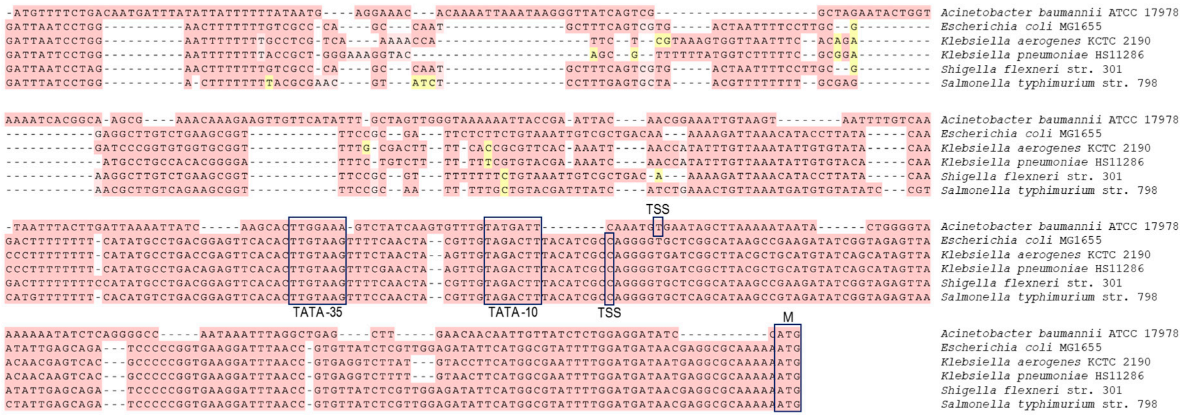

Figure S1. Promoter region of *OmpA* in *A. baumannii* ATCC 17978 and comparison with other Gram-negative bacteria. Based on this result, the *OmpA* promoters from six gram-negative bacteria were compared through the Multi-Align Program. The six strains of bacteria were *A. baumannii* ATCC 17978(Ab), *E. coli* MG1655(Ec), *Klebsiella aerogenes* KCTC 2190(Kaer), *Klebsiella pneumoniae* HS11286(Kpn), *Shigella flexneri* str. 301(Sfl), and *Salmonella typhimurium* str. 798(Styph). The location of the TATA boxes and TSS seemed to be conserved among the six bacteria. However, the *AbOmpA* promoter, including its TATA box and TSS, exhibited an atypical sequence, while the remaining five bacterial promoter regions showed a strong similarity.

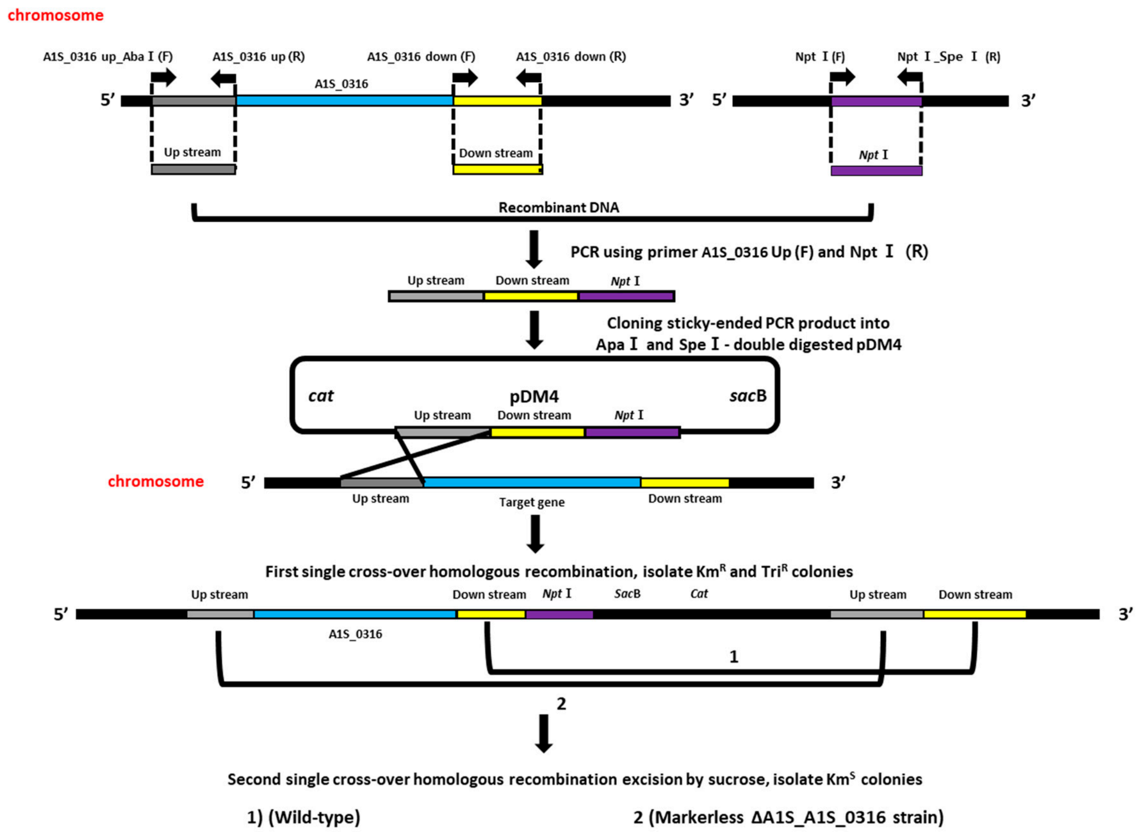

Figure S2. Schematic representation of the construction of the Δ*A1S_0316* mutant and its complementary strain of *A. baumannii* ATCC 17978.

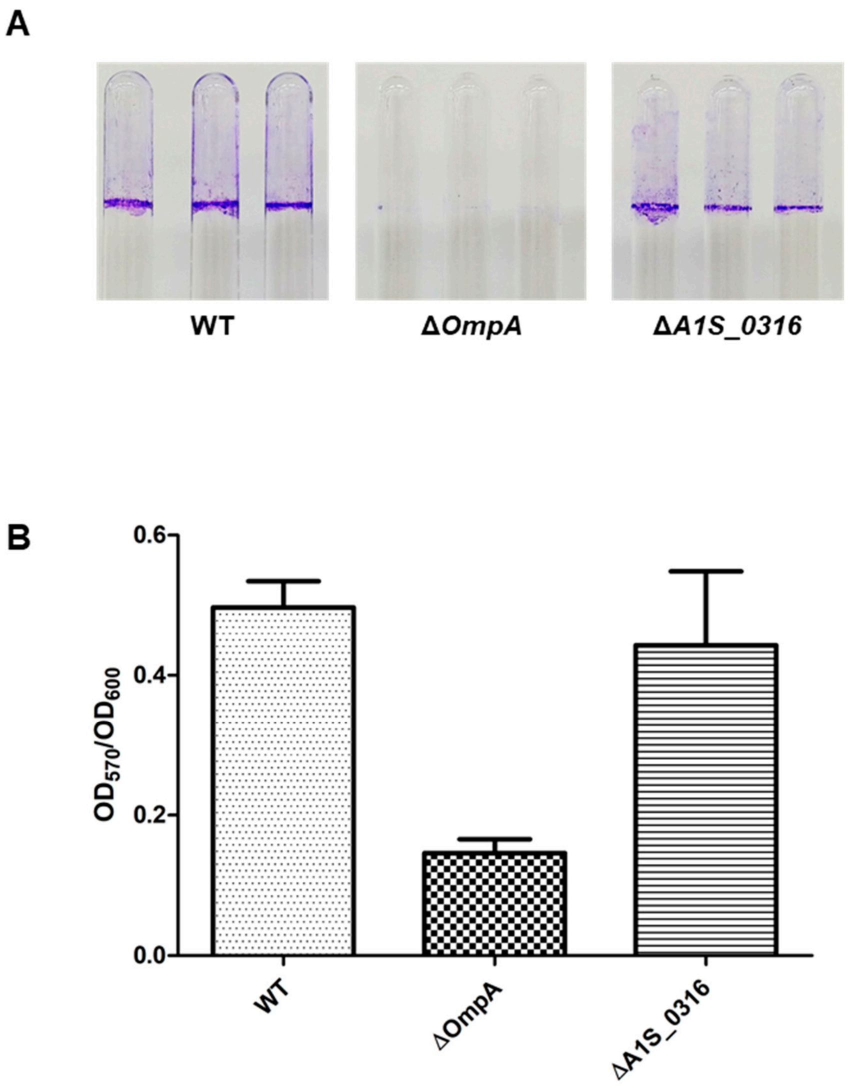

Figure S3. Qualitative assay showing biofilm formation on abiotic (glass) surface. Biofilms formed by *A. baumannii* ATCC 17978, Δ*OmpA* and Δ*A1S_0316* strains. Biofilms were allowed to form for 24 h at 37 °C in LB media without salt. (A) Determination of biofilm synthesis was performed using crystal violet staining. (B) Biofilm values (OD_{570}) were normalized by growth levels (OD_{600}) to compensate for differences in growth rate. Error bars represent standard deviations.

Table S1. Bacteria Strains, plasmids used in this study

Strain or plasmid	Relevant characteristic(s)*a*	Reference or source
**Bacterial strains**		
A. baumannii		
ATCC 17978	Prototype strain	[1]
ΔA1S_0316	ATCC 17978 with ΔA1S_0316	This study
ΔOmpA	ATCC 17978 with ΔOmpA	Laboratory collection
E. coli		
SY327 λ *pir*	*supE44 ΔlacU169* (ϕ80 *lacZ*ΔM15*) hsdR17 recA1 endA1 gyrA96 thi-1 relA1 λpir* (phage lysogen); plasmid replication	Laboratory collection
SM10 λ *pir*	*thi thr leu tonA lacY supE recA::RP4-2-Tc::Mu Km λpir* π-requiring plasmids; conjugal donor	[2]
DH5α	*fhuA2 lac(del)U169 phoA glnV44 Φ80' lacZ(del)M15 gyrA96 recA1 relA1 endA1 thi-1 hsdR17*	Laboratory collection [3]
BL21 star (DE3)	*F- ompT* $hsdS_B$ ($r_B^- m_B^-$) *gal dcm rne131* (DE3)	Laboratory collection
**Plasmids**		
pOH4	pHKD01 with *ompA* coding region with *nptI*; Km^R	[4]
pB4	derived from pET21a, HisTag-MBP, TEV cleavable	Laboratory collection
pB4::A1S_0316	pB4 with A1S_0316	This study

pDM4	Suicide vector; *ori*R6K sacBR, Cm^R	
pDM4::ΔA1S_0316	pDM4 with ΔA1S_0316::*nptI*; , $Cm^{R,}$ Km^R	This study
pAra::AbH-NS	derived from pBAD, AbH-NS *oriAb* cloned from pWH1266, Tc^R	Laboratory collection
pWH1266	pWH1277 cloned into pBR322, Amp^R, Tc^R	[5]
pWH1266::A1S_0316	pWH1266 with AbCRISPR-array	This study
pSA508	Promoterless, Amp^R	[6]
pSA508::AbOmpAp	pSA508 with AbOmpAp	This study

Table S2. Identified proteins that bind to *AbOmpAp*

Order	Size (kDa)	Proteins	Locus tag
①	154.9	DNA-directed RNA polymerase subunit beta	A1S_0288
②	61.1	30S ribosomal protein S1	A1S_1572
③	37.2	RNA polymerase alpha subunit	A1S_3056
	30.7	protein chain elongation factor EF-Tu	A1S_0279
④	29.3	Putative trancscriptional regulator	A1S_0316
	24.9	minC activating cell division inhibitor a membrane ATPase	A1S_0880
	19.0	putative two-component response regulator (citB)	A1S_3304
	14.2	putative universal stress protein A (UspA)	A1S_2692
⑤	12.5	putative DNA binding protein	A1S_0268
⑥	9.3	DNA-binding protein HU-beta	A1S_1637

Table S3. Oligonucleotides used in this study.

Primers	Primer sequence (5′→3′)
A1S_0316 SpeI UF	GTT GGG CCC TGC TGA GCA AAT TGG AAA AC
A1S_0316 UR	AAT AAG ACA TCA TGT AAA ATA ATA TTT TTG ATA ATT TAA AGT TTT CTT ACA C
A1S_0316 DF	TTA TCA AAA ATA TTA TTT TAC ATG ATG TCT TAT TCG TAT CCT T
A1S_0316 DR	CGA GGC AGA CTT ATA CAC AAT CAT TGC GGT TG
A1S_0316 NF	TGA TTG TGT ATA AGT CTG CCT CGT GAA GAA GGT G
A1S_0316 ApaI NR	GTT GGG CCC GAT CCG TCG ACC TGC AGG
A1S_0316 PstI F	TTC CAA TGC ATT GGC TGC AGG AGC TTA TTC CTC TGA GGG AG
A1S_0316 EcoRI R	GGA ATT CGA AAG AAG AGA CAT CGA GAA AGC
A1S_0316 LIC F	GGG CGG CGG TGG TGG CGG CAT GGC AAT TTC AAG TTT TGG C
A1S_0316 LIC R	GTT CTT CTC CTT TGC GCC CTA GAT CTC GCC CTT TTC TGA A
AbOmpAp EcoRI F	CCG GAA TTC AAC ACA AAA TTA AAT AAG GGT TAT CAG
AbOmpAp PstI R	GGT CTG CAG GAT AAC AAT TGT TGT TCA AGC TCA
AbOmpAp biotin F	CGA GTG TTA TAG TGA GCT CA
AbOmpAp biotin R	CAG CTA ATG GAG CAG CAA CA
AbOmpAp EMSA F	GGC AAG CGA AAC AAA GAA GTT G
AbOmpAp EMSA R	AAT GGA GCA GCA ACA AGC ATA G
16s rRNA-sense	GCA CAA GCG GTG GAG CAT
16s rRNA-antisense	CGA AGG CAC CAA TCC ATC TC
OmpA-sense	TTG CAC TTG CTA CTA TGC TTG TTG
OmpA-antisense	TGG CTG TCT TGG AAA GTG TAA CC

1. Baumann, P.; Doudoroff, M.; Stanier, R.Y. A Study of the Moraxella Group II. Oxidative-negative Species (Genus Acinetobacter). *Journal of Bacteriology* **1968**, *95*, 1520-1541.
2. Simon, R.; Priefer, U.; Pühler, A. A Broad Host Range Mobilization System for In Vivo Genetic Engineering: Transposon Mutagenesis in Gram Negative Bacteria. *Bio/Technology* **1983**, *1*, 784-791, doi:10.1038/nbt1183-784.
3. Taylor, R.G.; Walker, D.C.; McLnnes, R.R. E.colihost strains significantly affect the quality of small scale plasmid DNA preparations used for sequencing. *Nucleic Acids Research* **1993**, *21*, 1677-1678, doi:10.1093/nar/21.7.1677.
4. Kwon, H.I.; Kim, S.; Oh, M.H.; Na, S.H.; Kim, Y.J.; Jeon, Y.H.; Lee, J.C. Outer membrane protein A contributes to antimicrobial resistance of Acinetobacter baumannii through the OmpA-like domain. *Journal of Antimicrobial Chemotherapy* **2017**, *72*, 3012-3015, doi:10.1093/jac/dkx257.
5. Hunger, M.; Schmucker, R.; Kishan, V.; Hillen, W. Analysis and nucleotide sequence of an origin of DNA replication in Acinetobacter calcoaceticus and its use for Escherichia coli shuttle plasmids. *Gene* **1990**, *87*, 45-51, doi:10.1016/0378-1119(90)90494-c.
6. Choy, H.E.; Adhya, S. Control of gal transcription through DNA looping: inhibition of the initial transcribing complex. **1992**, *89*, 11264-11268, doi:10.1073/pnas.89.23.11264.
